# Incentives for Bushmeat Consumption and Importation among West African Immigrants, Minnesota, USA

**DOI:** 10.3201/eid2312.170563

**Published:** 2017-12

**Authors:** Emily Walz, David Wilson, Jacob C. Stauffer, Danushka Wanduragala, William M. Stauffer, Dominic A. Travis, Jonathan D. Alpern

**Affiliations:** University of Minnesota, Minneapolis, Minnesota, USA (E. Walz, W.M. Stauffer, D.A. Travis, J.D. Alpern);; African Career, Education, and Resources, Inc., Brooklyn Park, Minnesota, USA (D. Wilson);; Stillwater High School, Stillwater, Minnesota, USA (J.C. Stauffer);; Minnesota Department of Health, St. Paul, Minnesota, USA (D. Wanduragala)

**Keywords:** Global health, wild animals, emigrants, immigrants, zoonoses, public health, Liberia, travel medicine, hemorrhagic fever, Ebola, foodborne diseases, airports, food safety

## Abstract

The knowledge, attitudes, and practices surrounding bushmeat consumption and importation in the United States are not well described. Focus groups of West African persons living in Minnesota, USA, found that perceived risks are low and unlikely to deter consumers. Incentives for importation and consumption were multifactorial in this community.

Bushmeat hunting and butchery are risk factors for zoonotic disease transmission (*1*–*3*). However, less is known about health risks to those who consume products that are already butchered when purchased. Bushmeat in this report refers to meat from wild African animals such as rodents, hooved animals, carnivores, primates, and bats (*3*). 

Thousands of pounds of bushmeat are illegally imported into the United States annually (*4*), mostly from West Africa (*5*). A previous study of bushmeat consumption by African immigrants in the United States described mixed perceptions regarding the risks and benefits of consuming bushmeat (*5*). Improved understanding of the complex social drivers of these practices is needed to better characterize risk and formulate communication strategies.

To identify the cultural perspectives and knowledge, attitudes, and practices surrounding bushmeat importation and consumption, we held focus groups with members of the Liberian community living in the Minneapolis–St. Paul area of Minnesota, USA. Minneapolis–St. Paul has the largest Liberia-born population in the United States, and ranks fifth in overall African population in US metropolitan areas (*6*). Recognizing the history of stigmatization associated with increased risk for Ebola virus among persons from West Africa, we engaged a community-based organization to partner in the planning and execution of this study (*7*,*8*). Creating a comfortable environment where participants share personal experiences and insights freely is a key tenet of focus group methodology (*9*); this partnership was essential in gaining trust and maintaining cultural sensitivity.

Inclusion criteria for participant selection included: 1) minimum age 18 years, 2) self-identification as West African, and 3) willingness to discuss bushmeat in a group setting. The partner organization recruited community members by using a combination of purposeful sampling and social media advertisement and facilitated 3 focus groups (10–12 participants, each for 90 min) in January and February 2016; a designated research team member attended each session. A standard guide for questions was used for each session (Technical Appendix)**.** The University of Minnesota Institutional Review Board approved this study.

Sessions were audio recorded and transcribed; participants were not identified. Nonverbal cues (i.e., gestures, emotions, points of hesitation, nods of agreement) and other participant interactions were added to the transcript by a notetaker. We analyzed the collected data by using a modified grounded theory method with inductive analysis as previously described (*10*). Two authors (E.W., J.D.) analyzed each transcript by using an open and selective coding approach. Subsequently, all transcripts were analyzed together by using axial coding further describing relationships among themes (Table); representative quotes from participants were selected to exemplify a relationship or common theme (*9*) (Table). We supported validity of findings by using member-checking, triangulation of findings with multiple sources, and peer debriefing (*9*). Many themes were repeated in all groups; however, this study was limited by inability to confirm that we had reached saturation of perspectives. According to Creswell, it is ideal to repeat focus group sessions with new participants until novel perspectives no longer arise (*9*).

**Table:** Representative quotations and associated themes discussed by Liberian immigrants in bushmeat focus groups, Minnesota, USA*

Theme	Key quotation
1. Nostalgia/cultural connection is a driver for consumption	“So it goes back to the cultural thing, like she said. The taste and that which you're used to. I mean it's how you're brought up, and all that stuff. **It's just something like you go away to school and you just miss your mom's cooking.** So that's just what it is.”
2. Bushmeat is readily accessible and consumed when visiting friends/relatives in West Africa	Moderator: So for those that I hear, you know, about the regulations, about disease and all of that, do you think that if they were to go back home, would they still eat **bushmeat?** **“Oh yeah.”** **“Yeah.” (Many others nodding)**
3. Skepticism over potential zoonoses from bushmeat	“I don't believe that monkey or bat is carrying this virus. But these beliefs come from my experience. When I was growing up, I would talk to my grand uncle and we used to walk in the forest, teaching me how to survive in the forest… And he taught me one thing, anything that can kill any animal can kill you. And anything an animal carries that can kill it… When you see the animal, you’ll see it’s sick and you see it dead. So **anything that can kill me, the animal will not survive. So monkey cannot carry a virus that can kill me [and not look sick].”**
4. Cooking and proper food preparation can mitigate disease risk	“When you kill the bushmeat in Africa, before you even eat it, it goes over the fire, they dry the meat, and there it goes in the pot and we are cooking it in Africa—we are not cooking for five minutes. I **don’t care how the virus or bacteria is, when you put it in the fire it will not survive for a minute. When we start talking about Ebola, well, Ebola did not come from eating bushmeat, but the Ebola virus might have been on the meat, but when you put it on the fire, I don’t think that the Ebola virus could survive.”**
5. African bushmeat may be banned in the United States due to human health risks	“So if you tell somebody, you know someone who don’t know anything about Africa or West Africa and you tell a person, ‘I eat bushmeat,’ right, and they think ‘do you know how many animals over there… who have XYZ, difficult diseases?’ So, from their perspective, I’m going to freak out, like, **why are you bringing this into my country, where most likely, I don’t know what it carries, or it could be transmitted and there’ll be a big epidemic.”**
*Bold text indicates emphasis of quotation.	

Participants had resided in the United States from 6 months to 35 years; approximately half were female (Technical Appendix Table 1). All had consumed bushmeat, either abroad or in the United States. The 2 fundamental drivers of consumption in the United States were to 1) strengthen connection with African roots or 2) share the social experience with friends or relatives (Table). Many participants also reported frequent consumption of bushmeat while visiting West Africa (Table).

Most participants reported preference for what they described as “dried bushmeat.” “Drying” involved varying degrees of smoking, aging, and desiccation. Dried bushmeat, compared with raw or partially smoked products, was preferred for importation because its decreased odor is believed to reduce detection. 

Concern about zoonotic or foodborne disease dissuaded few participants from obtaining or consuming bushmeat, despite heightened awareness that wildlife could harbor Ebola virus. Among those who acknowledged this potential, most believed careful preparation and thorough cooking mitigated risk. For instance, participants cited traditional Liberian cooking techniques (extensive boiling for long durations) as a protective factor (Table). 

Some participants were knowledgeable of hunting and butchering techniques, but most participants purchased dried consumer products and had not participated in the processing of carcasses. Although there were consistent gaps in knowledge of import regulations, it was commonly perceived that political, public health, or discriminatory (e.g., racist, xenophobic) justifications were factors (Table).

These focus groups yielded detailed and nuanced information on the knowledge, attitudes, and practices related to bushmeat use and consumption among Liberians and Liberian Americans in a US metro area. Although this study did not directly enumerate the volume and type of bushmeat imported into the United States, our results provide a description of sociocultural factors involved on the demand side of the supply chain, a common gap in most risk assessments, and give insight into potential education and risk management strategies. We found that engaging the community in a culturally appropriate manner encouraged open dialogue, creating opportunities for education regarding import regulations and risk mitigation strategies (e.g., careful preparation and thorough cooking).

Technical Appendix Additional information for study of cultural perspectives and knowledge, attitudes, and practices surrounding bushmeat importation and consumption among members of the Liberian community living in the Minneapolis–St. Paul area of Minnesota, USA.
