# Supplementary material for: Incentives for Bushmeat Consumption and Importation among West African Immigrants, Minnesota, USA
Source: Emerg Infect Dis. 2017 Dec;23(12):2095–7. doi: 10.3201/eid2312.170563 (PMC5708257; doi:10.3201/eid2312.170563)
Supplement: Technical Appendix — Additional information for study of cultural perspectives and knowledge, attitudes, and practices surrounding bushmeat importation and consumption among members of the Liberian community living in the Minneapolis–St. Paul area of Minnesota, USA. [file 17-0563-Techapp-s1.pdf]

Article DOI: https://doi.org/10.3201/eid2312.170563

Incentives for Bushmeat Consumption and Importation among West African Immigrants, Minnesota, USA

Technical Appendix

Focus group question guide

1. Please tell us your name, how long you've lived in Minnesota, and what country you are from.

2. Can you describe what the term 'bushmeat' means to you?

• Probe 1: What other wild animal foods have you seen or heard of someone bringing back to the U.S. from West Africa?

• Probe 2: What medicines or other things do you take that are made with wild animal products?

3. What do people living in the U.S. from your community like about bushmeat?

4. What do people living in the U.S. from your community dislike about bushmeat?

5. How do you or people you know use or eat bushmeat in MN?

• Probe- are there other times people like to eat bushmeat in MN?

6. Can you describe for me how bushmeat gets to MN?

• Probe- is that process different than how you get bushmeat in West Africa?

7. It seems like getting bushmeat in MN is sometimes more complicated than just going to the supermarket. What are some of the important factors someone thinks about when deciding if bushmeat is worth this extra effort?

8. Is there anything else you'd like to mention on the topic we've discussed today?

Technical Appendix Table 1. Aggregated self-reported demographic characteristics of focus group participants.

Demographic characteristics of focus group participants (n = 32)	
Gender	
Male	44%
Female	56%
Age	
Minimum (years)	18
Maximum (years)	70+
Years residing in USA	
Mean (years)	13
Minimum (years)	0.5
Maximum (years)	35
Country of birth	
Liberia	97%
Sierra Leone	3%

Technical Appendix Table 2. Open and selective codes (with definitions) used in analysis of bushmeat focus group transcripts

Defining bushmeat	
Bushmeat is from the countryside	Bushmeat is animals hunted in countryside (rather than city)
Bushmeat is hunted animals	Bushmeat is a species that a hunter kills and brings back for food
Bushmeat is wild animals	Bushmeat is wild animals hunted from forest or bush
City vs forest animals as bushmeat	Wild animals hunted in cities might also be defined as bushmeat
U.S. 'domestic' bushmeat	U.S. wildlife (such as deer) is a type of 'domestic' bushmeat that I could eat
Food of rural poor	Eating bushmeat is associated with not having options and living in rural area in Liberia
Definition by species	Bushmeat can be defined as the following species
Cultural definition of bushmeat	The species defined as bushmeat or as a source of food varies by culture and perspective
Individual species preferences	Which species are defined as desirable foods may just be individual or local preference
Primitive	People may avoid bushmeat because it is seen as primitive or not Western
Stigma against talking about it	No one wants to talk about bushmeat, or people aren't talking about it because they emotional response to the subject
Taboo or religion influence	Eating some species is taboo for religious or cultural reasons
America as melting pot	U.S. culture and food is an amalgamation of things brought by different immigrant groups
Western perception of bushmeat	What Liberians think Americans or other 'Westernized' people think about bushmeat
Strong smell	Bushmeat products can have a strong/distinctive smell
Bushmeat for non-food purpose	Some African bushmeat species can also be used for non-food purposes
Drivers for consumption in U.S.	
Bushmeat is cheaper	In Africa, bushmeat is less expensive than domesticated meats
Good taste or smell	Positive sensory traits of bushmeat
Natural or organic	Bushmeat is desirable because it is natural
Supports health	Consuming bushmeat has special health properties
Tastes like home	Eating bushmeat reminds me of home and invokes connections with home
Wild taste	Preference for bushmeat because the taste is better than domesticated animal meat
Laws and regulations around bushmeat	
Educate doctors	Doctors need to know about how bushmeat is used; they're clueless
Legalizing bushmeat	If bushmeat could be tested or regulated, it could become legal
Liberian government	People don't trust the Liberian government to self-regulate meat for export
Regulations needed	Regulations are needed to ensure meat/foods are safe to eat
Ways bushmeat gets to the U.S.	
Airline luggage	You could bring bushmeat to the U.S. on a plane in airline luggage
Airline luggage- not anymore	One used to be able to bring in bushmeat in airline luggage, but not anymore
Available in town	Yes, African origin bushmeat is available in MN and I have eaten it
Not available in town	We don't eat bushmeat because it isn't available in MN
Buying bushmeat in African countries	Experiences purchasing bushmeat to bring back to U.S.
Get from friend or relative	Can acquire bushmeat in MN from friend or relative who had traveled home to Liberia
Postal service	You could mail bushmeat to the U.S. using the postal service
U.S. stores	You could purchase bushmeat at some U.S. stores
Zoonoses risk: skepticism and support	
Bushmeat has never caused disease	Experiences eating bushmeat for years and never had an issue
Confusion	Confusion or unsure if bushmeat has been linked to disease
Cooking cannot inactivate disease	Bushmeat could carry disease, and risks cannot be mitigated by cooking
Cooking inactivates disease	Bushmeat could carry disease, but risk can be mitigated by proper cooking
Food preparation	Food preparation and sanitation are key to preventing disease or illness when eating bushmeat
Worth the risk	If there is a risk to eating bushmeat, it is still worth it
Zoonosis origin stories	Explanations or examples about how disease has transferred from animals to people, usually focused on Ebola
Other themes	
Preparation of bushmeat	The ways to prepare bushmeat vary by preference
